# DiscovEpi: automated whole proteome MHC-I-epitope prediction and visualization

**DOI:** 10.1186/s12859-024-05931-2

**Published:** 2024-09-27

**Authors:** C. Mahncke, F. Schmiedeke, S. Simm, L. Kaderali, B. M. Bröker, U. Seifert, C. Cammann

**Affiliations:** 1 Friedrich Loeffler-Institute of Medical Microbiology-Virology, University Medicine Greifswald, 17475 Greifswald, Germany; 2 Research Unit Emerging Viruses, Leibniz Institute of Virology, 20251 Hamburg, Germany; 3 Institute of Immunology, University Medicine Greifswald, 17475 Greifswald, Germany; 4 Institute of Bioinformatics, University Medicine Greifswald, 17475 Greifswald, Germany; 5 Institute of Bioanalytics, University of Applied Sciences Coburg, 96450 Coburg, Germany

**Keywords:** MHC class I, Antigen presentation, Epitope prediction

## Abstract

**Background:**

Antigen presentation is a central step in initiating and shaping the adaptive immune response. To activate CD8^+^ T cells, pathogen-derived peptides are presented on the cell surface of antigen-presenting cells bound to major histocompatibility complex (MHC) class I molecules. CD8^+^ T cells that recognize these complexes with their T cell receptor are activated and ideally eliminate infected cells. Prediction of putative peptides binding to MHC class I (MHC-I) is crucial for understanding pathogen recognition in specific immune responses and for supporting drug and vaccine design. There are reliable databases for epitope prediction algorithms available however they primarily focus on the prediction of epitopes in single immunogenic proteins.

**Results:**

We have developed the tool DiscovEpi to establish an interface between whole proteomes and epitope prediction. The tool allows the automated identification of all potential MHC-I-binding peptides within a proteome and calculates the epitope density and average binding score for every protein, a protein-centric approach. DiscovEpi provides a convenient interface between automated multiple sequence extraction by organism and cell compartment from the database UniProt for subsequent epitope prediction via NetMHCpan. Furthermore, it allows ranking of proteins by their predicted immunogenicity on the one hand and comparison of different proteomes on the other. By applying the tool, we predict a higher immunogenic potential of membrane-associated proteins of SARS-CoV-2 compared to those of influenza A based on the presented metrics epitope density and binding score. This could be confirmed visually by comparing the epitope maps of the influenza A strain and SARS-CoV-2.

**Conclusion:**

Automated prediction of whole proteomes and the subsequent visualization of the location of putative epitopes on sequence-level facilitate the search for putative immunogenic proteins or protein regions and support the study of adaptive immune responses and vaccine design.

**Supplementary Information:**

The online version contains supplementary material available at 10.1186/s12859-024-05931-2.

## Background

The adaptive immune response is a complex and tightly regulated defence mechanism that protects the human host from a wide range of pathogens. It is triggered by the interaction of naïve T cells with antigen-presenting cells. In the case of CD8^+^ T cells, this requires the specific recognition of MHC-I-peptide complexes on the surface of antigen presenting cells by the clonotypic T cell receptors [1]. With the help of cytokines and co-receptors, this leads to activation and clonal expansion of the antigen specific cytotoxic CD8^+^ T cells and induces their differentiation into cytotoxic effector T cells (CTLs). These can migrate to the site of infection. When they re-encounter the same antigenic MHC-I-peptide complexes on the surface of infected cells, the CTLs induce apoptosis in the target cell through secretion of perforins, granzyme B and/or ligation of Fas on the surface of the target cells with Fas-ligand, which is expressed by the CTLs [2]. Most MHC-I-binding T cell epitopes (MHC-I-epitopes with the length of 8–12 amino acids) are generated through degradation of intracellular proteins by the ubiquitin–proteasome system (UPS) in the cytosolic compartment [3]. After their transport into the endoplasmic reticulum (ER), the peptides are loaded onto MHC class I molecules and the complexes reach the cell surface via the Golgi apparatus [1, 4]. To study the involvement of CD8^+^ T cells in the elimination of a pathogen, it is therefore necessary to analyse the generation of MHC-I-epitopes and to determine the peptide sequences. Such studies have been successfully performed for viral epitopes, e.g. from influenza A virus or SARS-CoV-2 [5, 6]. In addition, there is evidence that intracellular infection by bacteria such as *Listeria monocytogenes* or *Staphylococcus aureus* is accompanied by presentation of MHC-bound peptides [7, 8].

The knowledge of specific peptide sequences of epitopes can be used for the development of peptide-based vaccines which stimulate immune cells without the necessity of immunizing with the whole pathogen. This targeted approach enhances the efficiency in design and production of a vaccine, reduces possible side effects and increases the coverage if epitopes are shared between different strains of a pathogen [9]. One possibility for the development of peptide-based vaccines is based on the manual selection and extraction of candidate proteins [10] from databases like the UniProt Knowledgebase [11], followed by the prediction of single epitopes within each sequence by algorithms like NetMHCpan or MHCflurry [12, 13]. Further in-depth approaches considering the peptide-MHC-I-T cell receptor binding affinities such as TLImm [14] or PRIME [15] may provide higher accuracy in the prediction of possible neoantigens. However, these tools still rely on manually selected candidate sequences to be analysed. Pathogens like bacteria comprise complex proteomes containing a high number of proteins with diverse functions, cellular localization and interactions with the host cells. Analysing these for immunogenic epitopes necessitates either labour-intensive steps in manually choosing relevant candidate proteins or extensive computational resources and datasets which may lack abundancy. DiscovEpi focusses on the peptide presentation step and simplifies the prediction of immunogenic proteins or protein regions of whole proteomes by connecting the UniProt database with NetMHCpan to automatize the manual analysis. The automated extraction of species, strains, or even subcellular localization in combination with ranked epitope predictions based on epitope density and average binding score accelerates the search for promising candidates. Additionally, DiscovEpi visualizes all putative epitopes at their positions in the respective protein sequences. The colour intensity reflects their predicted immunogenicity, making the identification of immunogenic regions and fast comparison of potential vaccine targets easy and user-friendly.

### Implementation

The DiscovEpi algorithm is available on GitHub (https://github.com/cmahncke/DiscovEpi) containing packages for the usage under Windows 10 and 11 and Linux. DiscovEpi is implemented in Python 3.10 [16] and the Qt framework using PySide6 for integration (The Qt Company, Qt for Python project; PySide, max. version 6.6.3.1 available at https://pypi.org/project/PySide). Seaborn visualization library is used to produce the peptide map in the form of a heat map [17]. Remaining necessary third-party packages DiscovEpi has been built with are XlsxWriter (version 3.2.0), matplotlib (version 3.8.4), numpy (version 1.26.4), pandas (version 2.2.2) and requests (version 2.31.0). As interface between the UniProt database [11] and NetMHCpan [12] we implemented REST APIs hosted by UniProt and IEDB [18]. NetMHCpan (version 4.1) is implemented in the python scripts and executables. NetMHCpan generates a score of predicted strength of peptide-MHC binding, which is based on a trained neuronal network. This neural network calculates the strength of binding based on amino acid properties, peptide-MHC interactions and experimentally measured binding affinities [12]. This score is compared to the distribution of scores in a large, maintained reference library, and NetMHCpan provides a percentile rank as a measure of the relative strength of the predicted peptide-MHC-I binding. To date NetMHCpan proves to be the best predictor for MHC-I epitopes as it is the recommended predictor in the IEDB Analysis Resource [18]. The recommendation is updated based on weekly benchmarks which the authors of DiscovEpi will follow to ensure reliable results.

The first output file contains log data about the protein retrieval parameters and the protein data on a second sheet and is named “unp_ORGANISM_LOCATION.xlsx” where the bold letters are replaced by the respective input. After the epitope prediction step the retrieved putative epitopes with position normalized NetMHCpan binding score and the later described protein scoring metrics are written to the second sheet of the second output file named “nmp_ORGANISM_LOCATION_ALLELE.xlsx” in the previously specified directory with meta-data (UniProt-ID, Protein name, UniProt-Link) on the first sheet. The putative epitopes are ordered by their binding score showing the most promising ones in front. The file also contains data about the number of occurrences of each epitope and the query parameters.

### DiscovEpi epitope density and average binding score

DiscovEpi allows the protein-centric search for putative MHC-class I binding epitopes in whole proteomes based on the epitope density and average binding score of predicted epitopes. The binding score given by DiscovEpi is based on the percentile rank of each epitope as one of the output metrics from NetMHCpan. The percentile rank represents the relative binding affinity compared to a large reference group, including binding affinity scores for a diverse set of peptides and MHC class I molecules from experimental measurements and known MHC class I binding datasets. It is also used by DiscovEpi to discard peptides scoring higher than a specific percentile rank (default value = 3), assuming these peptides are non-binders [12]. To compare the epitopes in the resulting limited set of high affinity binders the DiscovEpi epitope score $s_{e}$ is computed by normalizing the percentile rank to values between 0 and 1 (Formula 1).

Formula 1: Epitope score. The percentile rank is normalized over the threshold (default value = 3). The difference between the threshold and percentile rank is divided by the threshold.

$$s_{e} = \frac{threshold - \% rank}{{threshold}}$$

The epitope score allows interpretation of protein sequences based on their density of potential epitopes. The epitope scores (Formula 1) are then used to compute the overall protein score for the whole protein sequence by averaging the retrieved epitope scores and over every possible epitope (Formula 2).

Formula 2: Protein score. $l_{p}$ is the protein sequence length and $l_{e}$ the epitope sequence length and $s_{e}$ the epitope scores of all retrieved epitopes for the respective protein sequence.

$$s_{p} = \frac{{\sum s_{e} }}{{l_{p} - l_{e} + 1}}$$

As the protein score $s_{p}$ does not differentiate between the presences of a few well binding or many weakly binding epitopes in a protein the epitope density is calculated as well (Formula 3). The epitope density $d$ of a protein defines the ratio of the number of predicted epitopes to the total number of possible oligopeptides of the same length in this protein. Thereby in combination with the protein score, a rational is created to value the length-independent immunogenicity of a protein so that proteins of different lengths can easily be compared. However, very short proteins can skew the output to give high epitope densities which will be visible in the epitope map and have to be handled with care during evaluation of the data.

Formula 3: Epitope density. $l_{s}$ is the protein sequence length and $l_{e}$ the epitope sequence length and $s_{e}$ the epitope scores for all MHC class I epitopes retrieved for the respective protein. Here, the score itself is not essential but the number of retrieved epitopes scoring below the threshold so that in combination with the protein density score (Formula 2) a holistic evaluation is possible.

$$d = \frac{{\# (s_{e} < threshold)}}{{l_{s} - l_{e} + 1}}$$

### DiscovEpi visualization

**Fig. 1:**
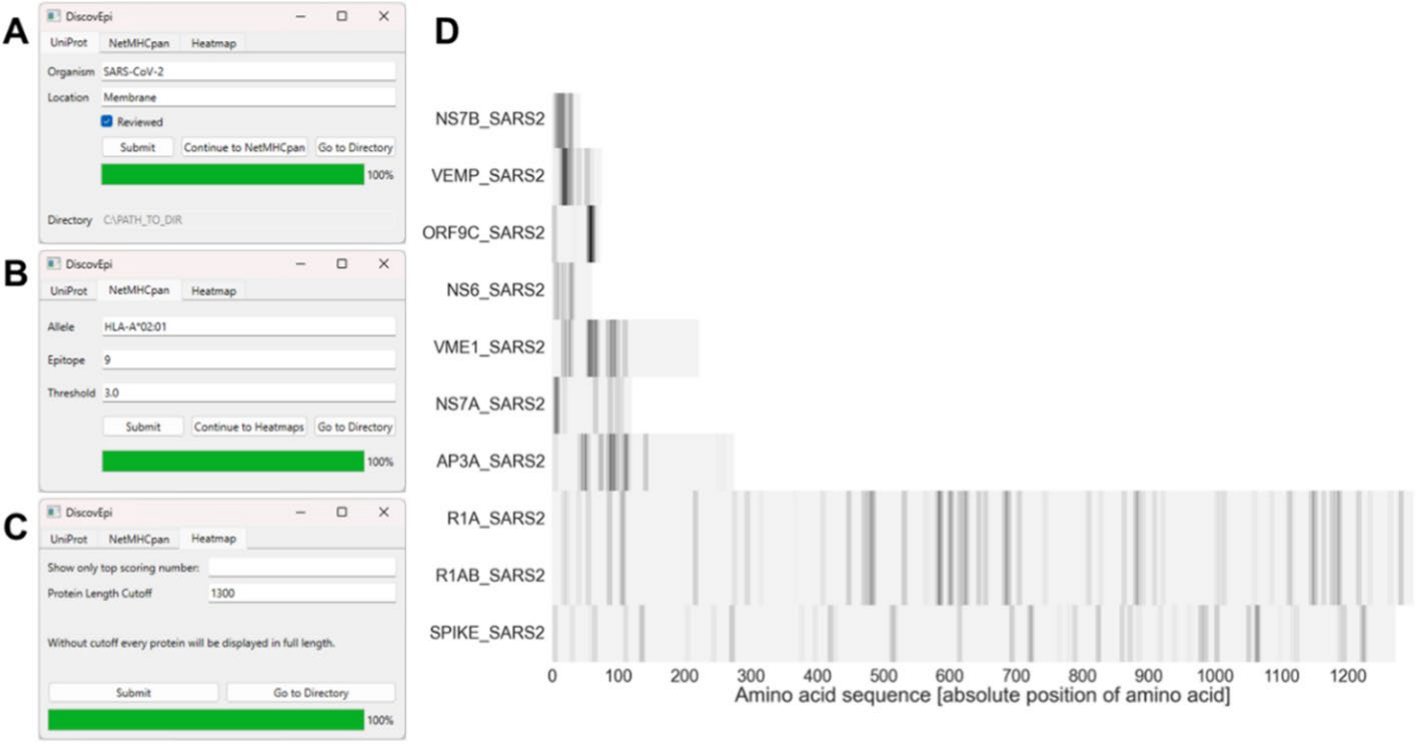
DiscovEpi schematic workflow and output. Workflow of DiscovEpi: **A** the protein sequence retrieval to generate the dataset; **B** the prediction parameters encompassing HLA allele, peptide length and NetMHCpan-score threshold; and **C** the parameters for visualization parameters of the predicted epitopes with the maximum number of top scoring proteins and the maximum protein length to be depicted. The resulting heat map (**D**) is generated with the parameters shown in **A**–**C** where each bar marks the presence of one or multiple overlapping putative epitopes and the intensity indicates the epitope score. The length of the protein is visualized through the light grey background. The proteins are ordered by their epitope density

The epitope map is created using a heat map with amino acid positions on the x-axis and the proteins on the y-axis. Each line of the heat map describes the amino acid sequence of one protein. By default, the length of the x-axis matches the length of the longest protein in the set. However, this value can be set individually as DiscovEpi takes the maximum length as input selected on the third tab of the he graphical user interface (GUI) (Fig. 1C). The length of shorter proteins is illustrated as light grey background which represents protein but no epitope whereas white background represents no protein so automatically no epitopes. Numerically, the underlying matrix contains the value 0.5 at each protein position and 0.0 when there is no protein. For each epitope of each protein the DiscovEpi scores are added to the default 0.5 in the respective line and amino acid sequence position (row). If there are overlapping epitopes the scores are added up. Visually, the intensity of the grey epitope markings depends on the calculated scores i.e., the darker the epitope marking, the higher the score. A high score here can reflect few very probable epitopes or many of less probability. Limiting the x-axis length increases resolution of shorter proteins since the shorter sequences are visualized using more horizontal space. Vertically, the resolution of the epitope map can be enhanced by setting a maximum number of proteins to be visualized on the map. This value can also be set on the third GUI tab (Fig. 1C). Especially bacterial protein sets can extend the resolution since the vertical height of the figure is fixed. The proteins shown on the map are ordered according to the DiscovEpi protein score so that even if the number is limited, the map still shows the most promising proteins. The resulting map is saved as PNG-file named “ORGANISM_LOCATION_heatmap.png” to the location specified on GUI tab one (Fig. 1A).

## Results

DiscovEpi is ready to use on the operating systems Windows (10/11) and Linux. Windows user download the “DiscovEpi_win” directory with the executable called “DiscovEpi_win.exe”. Linux users download the “DiscovEpi_linux” directory and run the python script “installation_gui.py” before running DiscovEpi with the file “main_gui.py”. The program requires a stable internet connection. A more comprehensive guide is provided as “README.md” in the Github repository.

### Workflow and functionality

DiscovEpi enables the automatic epitope prediction and extends the approach from single protein analysis to broad analysis of protein groups with a shared subcellular localization or even whole proteomes. To validate the described tool, we created a use case in which DiscovEpi has been used to identify epitope rich regions in membrane-associated proteins from SARS-CoV-2. The GUI of DiscovEpi (Fig. 1A–C) allows the automated extraction of all SARS-CoV-2 sequences with specific features. The text fields on the first tab specify the set of proteins to retrieve (Fig. 1A). The set of proteins must be selected for an organism or strain listed in the UniProt (https://www.uniprot.org/help/organism-name) and can be restricted for a subcellular location listed in the UniProt (https://www.uniprot.org/help/subcellular_location). Additionally, the set can be restricted to only reviewed database entries to ensure validity of the data.

The DiscovEpi output contains a Microsoft Excel spreadsheet with meta-data information about each protein, including UniProt ID, protein/gene name, length of the signal sequence (if available) and whole protein sequence, subcellular localization, and the amino acid sequence. DiscovEpi can automatically remove redundant sequences before the prediction step. The epitopes will be predicted for every protein sequence retrieved from the UniProt after setting the NetMHCpan parameters (Fig. 1B). DiscovEpi passes the following filter settings to NetMHCpan: the HLA allele (default = HLA-A*02:01), the peptide length (default = 9) and the threshold to limit predicted epitopes for the best scoring percentage by the NetMHCpan epitope percentile rank (default = 3). The default threshold is set higher than NetMHCpan’s current value to consider a peptide a weak binder to retrieve greater datasets including even weak binder for this quantitative approach and tolerate changes of the value in updated versions of NetMHCpan. Finding false-positive peptides is considered better than overlooking true epitopes. Based on the NetMHCpan predictions of single epitopes DiscovEpi calculates the epitope density and average binding score of the predicted epitopes in each protein sequence individually to assess the putative T cell epitope dependent immunogenicity. The epitope density describes the number of all putative epitopes in relation to all possible peptides of the respective length in the protein’s amino acid sequence. The average protein binding score considers only on the retrieved putative epitopes to gain information about the putative binding to MHC-class I without relation. For the protein-centric visualization of the peptides DiscovEpi allows the user to set up the visualization range (Fig. 1C) and to create a heat map of the dataset of protein sequences with their putative epitopes (Fig. 1D). The heat map is scaled on the maximum protein length (default is the length of the longest sequence in the set) and the number of top ranked protein sequences (default = 50). The proteins to be displayed on the map are ordered according to the calculated epitope density. Limiting the number of depicted proteins automatically sets a focus on the proteins with the highest epitope density.

IEDB hosts an epitope mapping visualisation method called Immunome Browser which incorporates a very similar pathogen selection process and epitope visualisation method [18]. However, Immunome Browser prediction is based on experimental data, thus showing already known epitopes based on data from T cell, B cell and MHC ligand assays, however the results e. g., for T cell epitopes from *Staphylococcus aureus* are sparse. In comparison to that DiscovEpi benefits by integrating NetMHCpan’s prediction method and contributes more to find novel epitope candidates for in vitro analysis.

### Use case: prediction of putative SARS-CoV-2 epitopes

For the use case we retrieved 10 unique reviewed protein entries associated with the organism SARS-CoV-2 and the localization term “membrane” (Fig. 1A, Supplementary Table 1). The corresponding membrane spanning regions were manually added to the list (Supplementary Table 2). Prediction for epitopes of the length “9” for the allele “HLA-A*02:01” ended up with a total of 521 peptides (threshold 3, Fig. 1B, Supplementary Table 2).

The protein length varied between 43 amino acids from the *NS7B_SARS2* to 7096 amino acids of the replicase *R1AB_SARS2* (Supplementary Table 1). For better resolution of the display we limited the sequence length to 1300 amino acids to still display the full glycoprotein *SPIKE_SARS2* which is already used in vaccines [19] at the expense of truncated sequences of the two replicase proteins *R1A_SARS2* and *R1AB_SARS2* (Fig. 1D). The resulting peptide map shows wide regions with numerous bands of average intensity which indicate sequences containing continuous and few overlapping peptides e. g. for the proteins *VEMP_SARS2* (position 4–66) and *AP3A_SARS2* (position 69–120). On the other hand, there are shorter dark grey and black bands indicating that these regions contain many overlapping or a few peptides with high epitope scores e. g. for the proteins *VEMP_SARS2* (position 16–24) and *ORF9C_SARS2* (position 57–65). In contrast, the spike protein *SPIKE_SARS2* contains regularly distributed epitope regions. Some of the predicted epitopes have been found to be recognized by T cells in COVID-19 patients. Saini et al*.* performed genome-wide T cell epitope mapping in COVID-19 patients. By analysing 10 HLA haplotypes the group identified 122 unique CD8 + T cell epitopes in the genome of SARS-CoV-2 [20]. For HLA-A*02:01, 19 of the 21 identified epitopes were also predicted by DiscovEpi with better than average scores. To validate the predictions, the immunogenicity and efficacy in vaccines of the predicted epitope regions need to be further investigated in vitro. As the results clearly indicate the immunogenic potential of a set of proteins and the sites of interest, we could compare these to other pathogens. Applying the same DiscovEpi parameters to the 5 membrane associated proteins of influenza A virus (strain Puerto Rico/8/1934 H1N1), we observed fewer epitope rich regions with lower binding scores (Supplementary Fig. S1). This was reflected by the epitope density as well. The mean epitope density of the SARS-CoV-2 protein set (0.108, Supplementary Table 2) is higher than the one of influenza A (0.044, Supplementary Table 3).

**Fig. 2:**
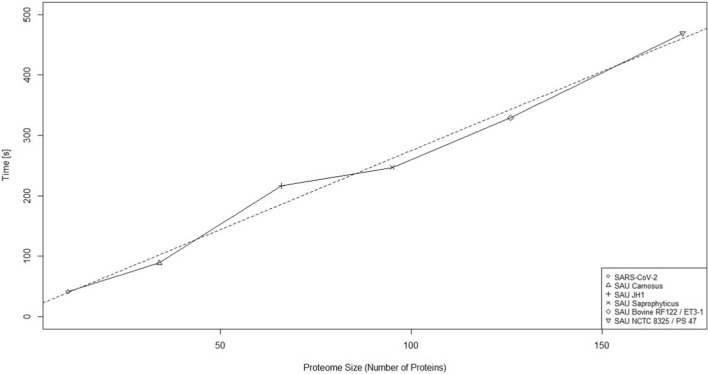
Runtime analysis of DiscovEpi. The time elapsed during epitope prediction was compared with the number of proteins in each proteome. Runtime analysis was performed with the Windows executable on Windows 11 with Intel Ultra 9 185H and 32 GB RAM. Sets of reviewed membrane associated proteins of SARS-CoV-2 and 5 strains of *Staphylococcus aureus* (SAU) were used to cover different proteome sizes. The dotted line describes the linear regression with a slope of 2.61 and *p*-value of 6.6e−5

Epitope prediction is the only step determining the runtime. Runtime analysis has been performed with the Windows executable on Windows 11, Intel Ultra 9 185H and 32 GB RAM with a stable internet connection. The analysis included protein sets for reviewed membrane associated proteins of SARS-CoV-2 and 5 strains of *Staphylococcus aureus* to cover a wide distribution of proteome sizes. Protein retrieval via UniProt only requires one request on the REST API which only takes one second independent of the proteome size. Equally, creating the epitope map takes one second. Epitope prediction time increases with the number of proteins in a linear manner since there is one request per protein posted to the NetMHCpan hosting server (Fig. 2). The linear regression indicates that per protein the epitope prediction step takes 2.61 s more time.

## Conclusion

DiscovEpi provides an in silico tool to assess the involvement of CD8^+^ T cells in the defence against specific pathogens, such as SARS-CoV-2 shown here. The tool reduces time and workload of epitope predictions for a set of proteins substantially and generates a comprehensive global view on putative immunogenic protein regions. Thus, DiscovEpi can serve as the first step in the search for new targets of epitope-based drugs and vaccines against pathogens.

### Outlook

To further improve DiscovEpi as a tool in T cell epitope analysis there are still features to include in the future. As proteins contain domains of more importance (receptor-binding domain) and less (transmembrane domain) it would be a benefit to visualize these domains in the epitope map. Furthermore, epitope-rich regions display a promising immunogenic potential and a function to identify and value such hotspots would improve the general protein score metric. It would also overcome DiscovEpi’s averaging out limitation in the protein score and let the user put emphasize on short immunogenic regions inside proteins.

Technically, DiscovEpi would improve from the local installation and integration of NetMHCpan, which is available for academic purpose. Finally, the inclusion of NetMHCIIpan to predict epitopes for MHC class II would extend the workflow and provide a more comprehensive analysis.

### Availability and requirements

The scripts of DiscovEpi is publicly and freely available via Github (github.com/cmahncke/DiscovEpi). All codes are written in Python3 and also available as stand-alone executables for the operating systems Microsoft Windows (tested for Windows 10 and 11) and Linux (tested for Ubuntu 24.04). The software is licensed as GNU GPL and there are no restrictions for academic or non-academic use.

## Supplementary Information

Supplementary file1

Supplementary file2

Supplementary file3

Supplementary file4

## Data Availability

DiscovEpi is freely available at GitHub (https://github.com/cmahncke/DiscovEpi). All information regarding its installation and application is provided.

## Abbreviations

MHC: Major histocompatibility complex
CTL: Cytotoxic T cells
UPS: Ubiquitin proteasome system
ER: Endoplasmic reticulum
GUI: Graphical user interface
HLA: Human leucocyte antigen
