# Supplementary material for: DiscovEpi: automated whole proteome MHC-I-epitope prediction and visualization
Source: BMC Bioinformatics. 2024 Sep 27;25:310. doi: 10.1186/s12859-024-05931-2 (PMC11438315; doi:10.1186/s12859-024-05931-2)
Supplement: Supplementary file 1 — Supplementary file1 [file 12859_2024_5931_MOESM1_ESM.docx]

# **DiscovEpi: Automatic whole proteome MHC-I-epitope prediction and visualization**

**Mahncke C, Schmiedeke F, Simm S, Kaderali L, Bröker B, Seifert U, Cammann C**

# content:

# Supplemetary Figure S1.


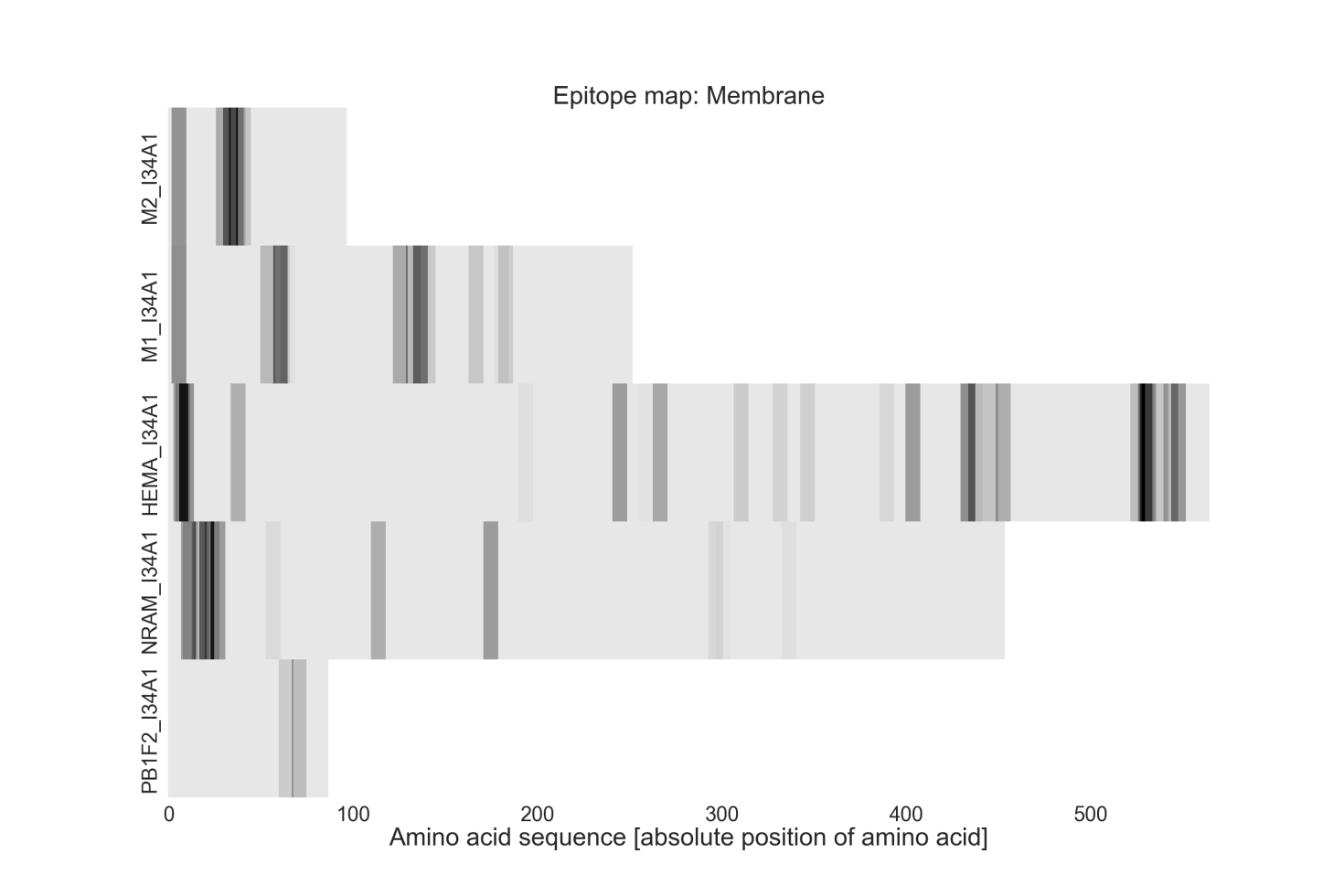


Supplementary Figure S1: Map of predicted epitopes for influenza A proteins with membrane association binding to HLA-A*02:01 restricted to peptides of nine amino acids.
